# A journey through follow‐up for neurodevelopmentally at‐risk infants—A qualitative study on views of parents and professionals in Liverpool

**DOI:** 10.1111/cch.12713

**Published:** 2019-07-30

**Authors:** Ayuko Komoriyama, Fauzia Paize, Esme Littlefair, Chris Dewhurst, Melissa Gladstone

**Affiliations:** ^1^ Department of Women and Children's Health, Institute of Translational Medicine, Alder Hey Children's NHS Trust University of Liverpool Liverpool UK; ^2^ Liverpool Women's NHS Foundation Trust Liverpool UK

**Keywords:** at‐risk infants, neonatal follow‐up, neurodevelopmental follow‐up, premature birth, views of parents

## Abstract

**Background:**

With improving neonatal intensive care, more preterm babies or those with hypoxic‐ischaemic encephalopathy are surviving the newborn period. These babies are at high risk of neurodevelopmental delay. No studies to date have looked at the views of parents and professionals in relation to the processes of follow‐up for these infants.

**Methods:**

We conducted a qualitative study in order to understand the views of parents of preterm babies or those with hypoxic‐ischaemic encephalopathy as well as the views of professionals who manage and support these families. Parents were recruited through general neonatal follow‐up clinics, neonatal nurse liaison services and community child health clinics and professionals through the neonatal unit and neurodevelopmental paediatrics services. We conducted in‐depth interviews using an open‐ended topic guide, which were audio recorded, transcribed and coded. We conducted a thematic content analysis where themes were inductively highlighted and grouped by consensus in order to conclude on major themes and subthemes.

**Results:**

Three major themes were identified for parents and professionals. These were the following: (a) What is the future, (b) What is the journey and (c) Who can help me? Parents wanted better information earlier about the prognosis and diagnoses through face to face, honest consultations with follow‐up information available on the Internet. The most important requirements for follow‐up clinics were honesty, reassurance, consistent pathways of follow‐up and the need for a lead professional in the process. Alongside the follow‐up process, there was a need for support groups and psychological support

**Conclusions:**

This study highlights the desire by parents for early information on the likely long‐term outlook for their babies but the need to ensure that the information and support, which is given, is provided appropriately and with consideration in order to provide the best care of the whole family.

**Key messages**

- Improved neonatal intensive care is leading to more infants surviving to discharge in neonatal units globally.
- There is lack of clarity as to what information and support parents and professionals feel would be most helpful when following‐up infants who are born at risk and discharged from a neonatal unit.
- Parents enunciated the need for clearer information about the future for their babies to be provided by professionals from the start—uncertainty is challenging for both parents and professionals to manage.
- Parents valued honest and consistent information provided face to face rather than through other modalities.
- Parental mental health is crucial to support not just during hospital but after discharge.

## 1 INTRODUCTION

In the United Kingdom, advances in medical care have led to improvements in the survival of babies born extremely premature (Costeloe et al., 2012) as well as for those babies with hypoxic‐ischaemic encephalopathy (HIE). Better care comes in the form of better ventilation protocols, improved nutritional support and early identification of infection for premature infants (Horbar et al., 2012). For sick term babies with HIE, outcomes have vastly improved with the advent of total body cooling (Jacobs et al., 2013). These children are at risk of poor neurodevelopmental outcome (Kumar et al., 2008; Moore et al., 2012).

The National Institute for Health and Care Excellence has recently published guidelines for the “Developmental follow‐up of children and young people born preterm” (National Institute of Clinical Excellence, 2017). The guidelines detail how clinicians should conduct neurodevelopmental follow‐up including enhanced support and surveillance for children and also provide guidance on how information and decisions should be shared with parents.

Follow‐up clinics provide a space for professionals to identify the infants with difficulties and refer them on to appropriate early intervention programmes so that support and treatment is given in a timely manner (Voller, 2018). We know that follow‐up services can have a positive impact on parents' mental health and family well‐being by providing families with a point of contact to seek advice, raise their concerns and be reassured (Shevell, Majnemer, Rosenbaum, & Abrahamowicz, 2001). Evidence is limited, however, as to the other benefits of these clinics and whether parents and professionals feel that the services on offer are effective.

A study from the United States observed parents during follow‐up clinics coupled with semi‐structured interviews that investigated parent perspectives on follow‐up (Hobbs, Tschudy, Hussey‐Gardner, Jennings, & Boss, 2017) Parents taking part in this study described how they found the lack of information prior to and during follow‐up clinics very difficult. Additionally, parents wished they could have more explanation of the examinations taking place during clinic appointments and more information on child development and parenting. This lack of information left parents feeling uncertain and vulnerable and unclear as to what they could do to enhance their child's care (Hobbs et al., 2017). There are no studies, however, that consider the overall journey for parents in terms of what parents and professionals see as most advantageous and supportive within their follow‐up care package as well as which issues cause most concern for caregivers.

In this study, we aim to gain information on the perceptions and views of parents and professionals who look after infants who are at high risk of neurodevelopmental delay who have been discharged from the neonatal intensive care unit (NICU) in a specific area of the United Kingdom (Merseyside). We aim specifically to explore where, how and who parents get their information from regarding development and future outcomes for their infants.

## 2 METHODS

Our aim was to understand the perceptions and the subjective experiences for parents and professionals who care for infants who are born with a high risk of neurodevelopmental delay and who are discharged from a neonatal unit. We chose qualitative methodology to undertake this enquiry, and we outline our methodology following the COREQ guidelines (Tong, Sainsbury, & Craig, 2007). Our epistemological position was of interpretative subjectivism where parents and professionals use real‐world descriptions and phenomena of their experience, but within this, our research team had an understanding that the world does not exist independently of our knowledge of it. With a position of relativism, we know that reality is subjective and differs from person to person and that this needs to be taken into account within our interpretation of our results (Scotland, 2012).

We chose in‐depth interviews with parents and professionals as our major method of data collection for a number of reasons. This included ease of recruitment and feasibility of data collection, providing us with a chance to gain more of a narrative perspective separately from each parent. Interviews also allowed anonymity and enabled parents to talk freely. This study was sponsored by the University of Liverpool. The study and *all documents used in the study were approved by the National Research Ethics Service Committee West Midlands (IRAS Project ID: 217848, REC reference 17/WM/0016)*.

### 2.1 Recruitment and sampling

The study took place in the Liverpool and Sefton areas of Merseyside. Merseyside has a population of 1,391,113. Liverpool has the highest population with 478,580 in the region, and Sefton has a population of 273,707.139 (2015 data). The Liverpool Women's NHS Foundation Trust (LWH) is a tertiary maternity hospital, which takes care of up to 1,400 neonates every year in their NICU. Alder Hey Children's Hospital (AHCH) is also a tertiary centre for paediatrics, which treats over 270,000 children every year.

We recruited two groups of participants for this study: parents and professionals (Table 1). In recruiting parents, we purposively identified mothers and fathers of babies who were born prematurely (less than 37 weeks) and mothers and fathers of babies who had HIE. We focused on parents (18 or over) who had children between the ages of 0 and 3 within the Merseyside region as we felt that parents of older children might have a historically different experience of the systems. We recruited those fluent in English to enable us to explore views and opinions in detail and minimise misunderstandings and miscommunications. Second, we recruited professionals working with those babies and families such as doctors, nurses and physiotherapists who have been involved in follow‐up of at‐risk infants in the Merseyside region. We recruited parents from the two base hospitals LWH and the community paediatric and neurodevelopmental follow‐up clinics, which are linked to AHCH. At LWH, nurses provided participants who were coming to neonatal follow‐up clinic and those on the community with an information leaflet and a brief description of the study. The primary investigator visited the neonatal ward to hand out information leaflets to parents of babies soon to be discharged. Community physiotherapists from the AHCH neurodevelopmental follow‐up services also were provided with information leaflets, which they used to introduce the study to parents. Professionals were contacted through ward meetings, posters in hospital rooms and through emails to all professionals in each department. If parents or professionals were interested in taking part in the study, they were then contacted to provide more information about the study and to ensure they were willing to take part.

**Table 1:** Sampling matrix and demographics of participants in the Foundation Study

	Babies age	Age of participant				
		18–35		36+		Total
		Male	Female	Male	Female	
Parent interviews:	0–6 months	3	2	2	1	8
	7–12 months	2	2	0	1	5
	13 months to 3 years	7	2	4	1	14
	Total	12	6	6	3	27
Professional interviews	Neonatal service	Neonatal nurse		2		11
		Consultant Neonatologist		2		
	Neurodevelopmental paediatrics service	Community paediatrician		2		
		Neurodisability consultant		2		
		Physiotherapist		4		
		Occupational therapist		1		

Through purposive sampling, we took into account a number of factors in our framework, which we felt might influence the views and perceptions of parents. This included gender of the parent, age of the parent and those at different time points in their journey after discharge from the neonatal unit. We aimed for at least 24–28 interviews with the knowledge that this would likely enable us to reach a point of saturation where participants were not adding new information and themes (Table 1).

### 2.2 Data collection and analysis

#### 2.2.1 Data collection

All interviews were audio recorded and transcribed by the primary researcher to be analysed on software NVIVO.

#### 2.2.2 Analysis

We used a stepwise approach to thematic content analysis (Braun & Clarke, 2006). This included familiarising ourselves with the data, generating initial codes and searching for themes, reviewing themes, defining and naming themes and then producing/writing a report. Two investigators coded transcripts and compared themes prior to deciding on a final list of themes with the whole research team.

## 3 RESULTS

A total of 27 parents were recruited. This consisted of 18 mothers and 9 fathers. Eleven professionals including community paediatricians, neurodisability consultants, neonatologists, physiotherapists, neonatal nurses and occupational therapists were interviewed.

Three major themes emerged from the analysis of transcripts from the interviews with parents and professionals. These are outlined in Table 2.

**Table 2:** Themes and subthemes which emerged from in‐depth interviews with parents and professionals on their views on the current neonatal and neurodevelopmental follow‐up services in Liverpool

Major theme	Subthemes
What's the future?	1. Information about future prognosis 2. Getting a diagnosis
What's the journey?	1. Consistency of follow‐up 2. Reassurance from a lead professional
Who can help me?	1. Information about support 2. Information from professionals

### 3.1 What's the future?

#### 3.1.1 Prior warning

Information about the future was of paramount importance to parents who were interviewed about the journey with their baby. One of the most common topics of conversation for parents related to how they were provided with information about the future for their child. This might have been, prior to birth, in the neonatal unit or after discharge either in the follow‐up clinic or in the community. Those parents who had antenatal complications such as premature rupture of membranes and who had prior warning of their impending delivery described how the opportunity to familiarise themselves with the NICU environment and staff was extremely helpful in enabling them to know what to expect in terms of the future for their baby *“I was explained about the NICU before I had them… someone came over early and went through everything from blindness, deafness and lung problems, everything he went through. Which was good. Obviously scary and worrying but he did go through everything.” FDNIDIPREM14 (mother)*.

#### 3.1.2 Uncertainty

The majority of the parents described a sense of frustration of not being provided clearer information earlier about the prognosis for their child and in some cases, parents of children who actually had had a diagnosis of cerebral palsy or autism expressed anger at the process of diagnosis—particularly the regret in not having had an earlier diagnosis or at least a discussion about these conditions with professionals. The uncertainty of a reassuring start which then changed to a poorer prognosis was the most challenging situation for parents: *“I just feel very much they (professionals) just don't know as much as we don't know.” FDNIDIHIM1 (father)*.

Some parents might be told that their child was fine at the time of discharge, but later on, their child developed cerebral palsy. Thinking retrospectively, parents enunciated the painfulness that a “false hope” can bring and many described how this led them to a lack of trust in professionals; *“… he did tell us what we should know but he was negative and I didn't want to see him again. Whereas other doctors would be probably overly positive. Not giving me the full understanding… it will be fine, it will be fine. Whereas Dr X just said he is not fine because of this, this is cerebral palsy…” FDNIDIHIF1 (father)*.

#### 3.1.3 “Still catching up”

Most parents professed that they were not aware of what the long‐term outlook was for their baby and did not recall having a conversation about milestones and neurodevelopment with professionals. One mother describes how her child had a delay in his speech but that she had not been aware as to what the normal milestones were and that she had not noticed the delay; *“… to be honest I didn't know there was something wrong when he wasn't talking. I didn't know.” FDNIDIPREM8 (mother)*. What parents understand as the likely risks and future prognosis for their babies varied considerably between the parents we interviewed. For example, second‐time mothers had better understanding of what could be different in premature babies; *“Obviously being the second time premy mum it was little bit easier …I don't think you were fully prepared for developmental delay… But I think that it should be an area that is discussed a little bit more…” FDNIDIPREM10 (mother)*.

#### 3.1.4 Clear information

Some parents described their feeling of depression and hopelessness when they were told that their child's prognosis—that their child might not be able to walk or talk. Parents expressed a clear desire to gain information about future prognosis in a way that was clear and as definite and realistic as possible. At the same time, one father described how important it was to concentrate on the strengths of his child, not just the weaknesses; *“it is hard of course, you are told he probably wouldn't walk, wouldn't talk. Even if you are just faced with that bang bang bang… all at once, you would just fall apart. But if we come down the line 2 years of X, he isn't walking, he is not talking and he doesn't eat but he smiles and he laughs and he doesn't stop smiling and laughing so interactive and he tries his best with everything. He is dead sociable. I think if we were told more along that side. Just the grey… you are not just given well they could do this not … not the case of probably they wouldn't do that.” FDNIDIHIM1 (father)*.

Professionals commented on the dilemma between providing clear information on a poor prognosis with the hope and acceptance that they want to give parents. It is that balance between enabling parents to enjoy a baby that they have waited for and providing them with the stark reality of what the future might hold; *“...I think it is a fine balance that I think if they continue what they are looking, they feel like they need tick boxes and that can impact on their enjoyment of their child...” FDNIDIPDN2 (professional)*.

Reassuring words are common from professionals who want to help and want to provide hope. At follow‐up visits, parents seek reassurance on the growth and development of their baby, for example, whether the baby is smiling, walking and talking. Ultimately, parents wanted to gain confirmation that the child was ok and that they were doing the right thing. *“I was always reading up in terms of his development because he was premature and I wanted to do everything that sort of also reassured myself that everything he was doing was normal.” FDNIDIPREM6 (mother)*.

### 3.2 What's the journey?

#### 3.2.1 Screening and identifying developmental delay

Both parents and professionals stated how unclear referral pathways and inconsistency in screening processes were major barriers for supporting these babies. In at least instances, worrying signs were identified by a variety of professionals (GPs, health visitors, physiotherapists or neonatologists), but the wait then to see a developmental paediatrician could be long; *“I had a child who was two who obviously had cerebral palsy but he wasn't referred until the age of 2.” FDNIDIPPT1*.

#### 3.2.2 Clinic follow‐up

Parents felt that neonatologists understood the course of their journey, and parents found that comforting*; “I like the clinic, because it has given me reassurance...” FDNIDIPREM5 (mother)*. However, not all parents felt the need for neonatal follow‐up. This may have been due to there being little information provided before discharge or in the invitation letter to the clinic as to why they were returning and what would happen in the clinic. Many parents felt that having one person who was leading their child's care was incredibly helpful with many identifying neonatologists as the lead professional particularly as they may have started the journey with parents and so were a familiar face at follow‐up*; “they (neonatologists) have a helicopter view and are able to then access physio, ophthalmology and community paediatricians. I like that she's seen in the clinic.” FDNIDIPREM6 (mother)*.

#### 3.2.3 Waiting for intervention

Many parents described the referral process from neonatal to paediatric services as a hurdle in their follow‐up pathway. Long waiting times can be detrimental to young infants with infants missing out on progressing with developmental milestones and professionals missing a window of opportunity to support and potentially enhance a child's neurodevelopment; “...*I think with little babies, especially with premature babies, have a window of development time, sometimes the waiting list is that window, 3 months is a long time in babies' development. FDNIDIPREM6 (professional)*.

Parents were reassured by having appointments with paediatricians as they felt as if the child was getting a “full MOT.” Some parents found community paediatric appointments overwhelming as they conducted a very thorough developmental assessment and sometimes mentioned potential diagnoses without warning. Some specialist neurodisability doctors made it clear that referring earlier to their services might prevent complications in the longer term. They felt that they were in a better place for following‐up high‐risk children particularly due to their close working with support systems in the community; *“there is no criticism to anyone but its more about you pick them up quite late and some of them have already developed changes with their posture or tone and they have not been seen by physios, so you already missed six to nine months of care for this child” FDNIDIPDCN2 (professional)*.

#### 3.2.4 Community support

Other parents identified physiotherapists as a lead professional, often as being very proactive and providing a realistic and holistic approach with sufficient information on developmental delay and cerebral palsy. Physiotherapists were also the main sources of information regarding other therapies such as sensory classes or hydrotherapy, support services, nurseries and schools in the community. Many mothers had had regular follow‐up health visitors in the community, and they felt well supported. However, some parents felt that they were not very knowledgeable about premature babies and they had limited understanding of the difficulties faced by mothers of premature babies in breastfeeding. For many parents, the first encounter that parents had with receiving information on developmental milestones was when the community neonatal nurses visited for the first time after discharge. They provided parents information of how premature babies' milestones can be different from term babies.

### 3.3 Who can help me?

Lack of support and advice left some parents feeling alone with their child who may have difficulties. One parent who had a child with global developmental delay said that she did not know that she could call to organise a health visitor review and was unaware of the way their services worked. She suggested that the hospital should make a list of support networks for parents*; “if I had known that there were all these different people who could help me I would have tried to ring them… but because I didn't know there is anything like that, I was trying to do it on my own.” FDNIDIPREM8*. Parents felt that generic support or parenting groups in community settings were focused only on “normal” term babies. Parents of infants born with HIE similarly found they did not quite fit in with a lot of support and awareness of prematurity in hospital and community settings but that they lacked support specifically for their children with HIE; *“HIE mums always say to me … ..everything is focused on premature babies. It's like they were kind of forgotten about … not unwelcome but like we were out of place.”* (*FDNIDIHIM1*).

The Internet was used by many parents but came with its own difficulties. Some families described the terrifying experiences online particularly if they had not been directed to specific sites; “*my instinct was to Google. And Google is such a scary forum”* (*FDNIDIPREM5*). Some parents used more specific sites—sometimes because they had been directed to them by professionals. Although parents described how they might have preferred to avoid searching online, sometimes, insufficient information was communicated during consultations, and then this was the only way to understand what the future risks and options might be for their child. Numerous parents described their use of social media with some finding it really helpful as other family's experiences were easier to relate to, but the feedback from social media enabled the option of actually sharing experiences of situations and becoming part of a community and support network. For a couple of parents, the knowledge gained from other parents who might be in more difficult situations with children with the same condition—the “worst‐case scenarios” was very hard. Parents expressed how they wished they could get the same amount of information from professionals as they did through the Internet and social media; “*Everything. Everything I know, I've got it through there. Everything. It's just amazing… But, should you really be getting that information from a private group? It should come from medical experts…” FDNIDIPREM4 (mother)*.

## 4 DISCUSSION

This is the first study that has explored the views of parents and professionals in the follow‐up of babies with a high risk of developmental delay. Parents do not just want to be “passive recipients for information” as has been highlighted in other studies (Young Seideman et al., 1997) but that they want to know facts and information that can help them identify when and where to look for more help and support for their baby.

Although many parents in this study described that they received an enormous amount of information whilst in the NICU and after discharge, many reported that they received an inadequate amount of information on the long‐term prognosis. Although it was perceived as heartbreaking and parents were not happy to be told negative information, parents appreciated an honest and realistic approach by neonatologists as previously suggested in other studies (Young Seideman et al., 1997), and in fact, parents who have knowledge about the outcomes of a premature birth often cope better with the stressful situation of having a premature baby (Granrud, Ludvigsen, & Andershed, 2014).

Some parents mentioned how they found some professionals reluctant to give detailed information about the future as the future was so uncertain. For professionals, some of this relates to the clinical difficulties of being sure about diagnoses early in the developing child's life. This dilemma is understandable but might be lessened if a link worker who works with teams that support parents of children with cerebral palsy was present very early on for parents. It certainly makes a case for the need and use of better prognostic tools, which might support clinicians in knowing from very early on which path to lead parents down.

Interestingly, some physiotherapists in this study were concerned that doctors sometimes gave a diagnosis too early when it was uncertain, damaging the relationship between parents and doctors. Studies from the United States have demonstrated in one study how the majority of paediatricians felt that an established diagnosis was important when making a referral to early intervention (Rasmussen, Racine, & Bell, 2016). Neurodisability professionals wanted to receive referrals as soon as a child developed signs so that they could refer further for more detailed assessments and investigation and prioritise interventions. Professionals, understandably, are often concerned that they will make the most suitable predictive judgements as to which children will or will not go on to have more major difficulties in the future (Rasmussen et al., 2016). It may be that using tools which are more predictive of cerebral palsy at an earlier stage, may help with this. Tools such as the Prechtl's General Movements assessment (Kwong, Fitzgerald, Doyle, Cheong, & Spittle, 2018), the Hammersmith Infant Neurological Exam (Mercuri et al., 1999) or more specific and sensitive developmental assessment measures (Wong, Santhakumaran, Cowan, & Modi, 2016) may be helpful in providing this prediction and should possibly be considered for use more routinely within services to support clinical predictions and enable families and children to be channelled appropriately from an earlier stage.

Many parents in our study clarified that they would like to receive more written information from professionals to take home to prevent the dilemma of searching online for unfiltered information that may be inappropriate and perceived as “scary” and “the worst thing” by some of the parents in our study. Parents remembered being told specific information such as that related to feeding support or the results of ultrasound head scans—particularly as some of these pieces of information may have been more shocking and distressing for parents (Woodward et al., 2014) particularly when they are in a stressed state with urgent medical issues ensuing. Parents did not remember being provided with more specific information about “what to expect in the future and what to look out for” maybe because it was done in a less distressed situation or maybe because what was provided or said was limited.

Integration of services is crucial in enabling families to feel supported and to gain the information that is needed with a multidisciplinary team approach. Linked professional working (paediatricians, physiotherapists, speech and language therapists, occupational therapists, clinical psychologists, audiologists, play therapists and hospital social workers) is likely to help children reach their developmental potential by intervening from an early age. Linking a service like this much more closely with the neonatal community nurses and health visitors would mean that children could more seamlessly be referred. It was clear from our results that a consistent follow‐up service for the child and family is crucial, particularly for those children who have been provided with a diagnosis and who need extra support. This type of service allows for a strong relationship between professionals and parents and enables a space for parents to ask questions or raise concerns.

This study specifically aimed to understand the views of parents and professionals with regard to neonatal neurodevelopmental follow‐up. We have gained a deeper understanding of this area, which we can focus future modelling of services upon.

## 5 CONCLUSION

Preterm babies and those who have suffered HIE are vulnerable to developmental delay. This study has provided a detailed insight into the views from parents and professionals as to what information and support is useful, helpful and necessary for families of infants at risk of neurodevelopmental delay.

## References

[cch12713-bib-0001] Braun, V. , & Clarke, V. (2006). Using thematic analysis in psychology. Qualitative Research in Psychology, 3(2), 77–101. 10.1191/1478088706qp063oa

[cch12713-bib-0002] Costeloe, K. L. , Hennessy, E. M. , Haider, S. , Stacey, F. , Marlow, N. , & Draper, E. S. (2012). Short term outcomes after extreme preterm birth in England: Comparison of two birth cohorts in 1995 and 2006 (the EPICure studies). BMJ, 345, e7976 10.1136/bmj.e7976 23212881 PMC3514472

[cch12713-bib-0003] Excellence, N. I. f. H. C. a. C. (2017). Developmental follow‐up of children and young people born preterm NICE guideline 72. Retrieved from London: https://www.nice.org.uk/guidance/ng72

[cch12713-bib-0004] Granrud, M. D. , Ludvigsen, E. , & Andershed, B. (2014). Parents' experiences of their premature infants' transportation from a University Hospital NICU to the NICU at two local hospitals. Journal of Pediatric Nursing, 29, e11–e18. 10.1016/j.pedn.2014.01.014 24582644

[cch12713-bib-0005] Hobbs, J. E. , Tschudy, M. M. , Hussey‐Gardner, B. , Jennings, J. M. , & Boss, R. D. (2017). “I don't know what I was expecting”: Home visits by neonatology fellows for infants discharged from the NICU (Vol. 44, pp. 331‐336). 10.1111/birt.12301 28833441

[cch12713-bib-0006] Horbar, J. D. , Carpenter, J. H. , Badger, G. J. , Kenny, M. J. , Soll, R. F. , Morrow, K. A. , & Buzas, J. S. (2012). Mortality and neonatal morbidity among infants 501 to 1500 grams from 2000 to 2009. Pediatrics, 129(6), 1019–1026. 10.1542/peds.2011-3028 22614775

[cch12713-bib-0007] Jacobs, S. E. , Berg, M. , Hunt, R. , Tarnow‐Mordi, W. O. , Inder, T. E. , & Davis, P. G. (2013). Cooling for newborns with hypoxic ischaemic encephalopathy. Cochrane Database of Systematic Reviews, 1, CD003311 10.1002/14651858.CD003311.pub3

[cch12713-bib-0008] Kumar, P. , Sankar, M. J. , Sapra, S. , Agarwal, R. , Deorari, A. K. , & Paul, V. K. (2008). Follow‐up of high risk neonates. Indian Journal of Pediatrics, 75(5), 479–487. 10.1007/s12098-008-0075-9 18537010

[cch12713-bib-0009] Kwong, A. K. L. , Fitzgerald, T. L. , Doyle, L. W. , Cheong, J. L. Y. , & Spittle, A. J. (2018). Predictive validity of spontaneous early infant movement for later cerebral palsy: A systematic review. Developmental Medicine and Child Neurology, 60(5), 480–489. 10.1111/dmcn.13697 29468662

[cch12713-bib-0010] Mercuri, E. , Guzzetta, A. , Haataja, L. , Cowan, F. , Rutherford, M. , Counsell, S. , … Dubowitz, L. (1999). Neonatal neurological examination in infants with hypoxic ischaemic encephalopathy: Correlation with MRI findings. Neuropediatrics, 30(2), 83–89. 10.1055/s-2007-973465 10401690

[cch12713-bib-0011] Moore, T. , Hennessy, E. M. , Myles, J. , Johnson, S. J. , Draper, E. S. , Costeloe, K. L. , & Marlow, N. (2012). Neurological and developmental outcome in extremely preterm children born in England in 1995 and 2006: the EPICure studies. BMJ, 345, e7961 10.1136/bmj.e7961 23212880 PMC3514471

[cch12713-bib-0012] Rasmussen, L. A. , Racine, E. , & Bell, E. (2016). A qualitative study of physician perspectives on prognostication in neonatal hypoxic ischemic encephalopathy. Journal of Child Neurology, 31(11), 1312–1319. 10.1177/0883073816656400 27377309

[cch12713-bib-0013] Scotland, J. (2012). Exploring the philosophical underpinnings of research: Relating ontology and epistemology to the methodology and methods of the scientific, interpretive, and critical research paradigms. English Language Teaching, 5(9). 10.5539/elt.v5n9p9

[cch12713-bib-0014] Shevell, M. I. , Majnemer, A. , Rosenbaum, P. , & Abrahamowicz, M. (2001). Profile of referrals for early childhood developmental delay to ambulatory subspecialty clinics. Journal of Child Neurology, 16(9), 645–650. 10.1177/088307380101600904 11575603

[cch12713-bib-0015] Tong, A. , Sainsbury, P. , & Craig, J. (2007). Consolidated criteria for reporting qualitative research (COREQ): A 32‐item checklist for interviews and focus groups. International Journal for Quality in Health Care, 19(6), 349–357. 10.1093/intqhc/mzm042 17872937

[cch12713-bib-0016] Voller, S. M. B. (2018). Follow‐up care for high‐risk preterm infants. Pediatric Annals, 47(4), e142–e146. 10.3928/19382359-20180325-03 29668022

[cch12713-bib-0017] Wong, H. S. , Santhakumaran, S. , Cowan, F. M. , & Modi, N. (2016). Developmental assessments in preterm children: A meta‐analysis. Pediatrics, 138(2), 1–12. 10.1542/peds.2016-0251 27471220

[cch12713-bib-0018] Woodward, L. J. , Bora, S. , Clark, C. A. C. , Montgomery‐Hönger, A. , Pritchard, V. E. , Spencer, C. , & Austin, N. C. (2014). Very preterm birth: Maternal experiences of the neonatal intensive care environment. Journal Of Perinatology: Official Journal Of The California Perinatal Association, 34(7), 555–561. 10.1038/jp.2014.43 24651730 PMC4154363

[cch12713-bib-0019] Young Seideman, R. , Watson, M. A. , Corff, K. E. , Odle, P. , Haase, J. , & Bowerman, J. L. (1997). Parent stress and coping in NICU and PICU. Journal of Pediatric Nursing, 12(3), 169–177. 10.1016/S0882-5963(97)80074-7 9198340

